# Prevalence and determinants of osteoporosis in patients with type 1 and type 2 diabetes mellitus

**DOI:** 10.1186/1472-6823-14-33

**Published:** 2014-04-11

**Authors:** Gudrun Leidig-Bruckner, Sonja Grobholz, Thomas Bruckner, Christa Scheidt-Nave, Peter Nawroth, Jochen G Schneider

**Affiliations:** 1 Practice for Endocrinology and Nuclear Medicine, Brückenstraße 21, Heidelberg 69120, Germany; 2 Department of Internal Medicine, Endocrinology and Metabolism, University of Heidelberg, INF 410, Heidelberg 69120, Germany; 3 Institute for Medical Biometry and Informatics, University of Heidelberg, INF 305, Heidelberg 69120, Germany; 4 Robert Koch Institute, Department of Epidemiology and Health Monitoring, General-Pape-Straße 62-66, Berlin 12101, Germany; 5 Luxembourg Centre for Systems Biomedicine (LCSB), Université du Luxembourg & Internal Medicine II, Saarland University Medical Center at Homburg/Saar, Kirrbergerstrasse 100, Homburg/Saar 66421, Germany

**Keywords:** Bone mineral density, Diabetes mellitus, Fractures, Osteoporosis, Vascular complications

## Abstract

**Background:**

Increased risk of osteoporosis and its clinical significance in patients with diabetes is controversial. We analyze osteoporosis prevalence and determinants of bone mineral density (BMD) in patients with type 1 and 2 diabetes.

**Methods:**

Three hundred and ninety-eight consecutive diabetic patients from a single outpatient clinic received a standardized questionnaire on osteoporosis risk factors, and were evaluated for diabetes-related complications, HbA1c levels, and lumbar spine (LS) and femoral neck (FN) BMD. Of these, 139 (71 men, 68 women) type 1 and 243 (115 men, 128 women) type 2 diabetes patients were included in the study. BMD (T-scores and values adjusted for age, BMI and duration of disease) was compared between patient groups and between patients with type 2 diabetes and population-based controls (255 men, 249 women).

**Results:**

For both genders, adjusted BMD was not different between the type 1 and type 2 diabetes groups but was higher in the type 2 group compared with controls (p < 0.0001). Osteoporosis prevalence (BMD T-score < −2.5 SD) at FN and LS was equivalent in the type 1 and type 2 diabetes groups, but lower in type 2 patients compared with controls (FN: 13.0% vs 21.2%, LS: 6.1% vs 14.9% men; FN: 21.9% vs 32.1%, LS: 9.4% vs 26.9% women). Osteoporosis prevalence was higher at FN-BMD than at LS-BMD. BMD was positively correlated with BMI and negatively correlated with age, but not correlated with diabetes-specific parameters (therapy, HbBA1c, micro- and macrovascular complications) in all subgroups. Fragility fracture prevalence was low (5.2%) and not different between diabetes groups. Fracture patients had lower BMDs compared with those without fractures; however, BMD T-score was above −2.5 SD in most patients.

**Conclusions:**

Diabetes-specific parameters did not predict BMD. Fracture occurrence was similar in both diabetes groups and related to lower BMD, but seems unrelated to the threshold T-score, <−2.5 SD. These results suggest that osteoporosis, and related fractures, is a clinically significant and commonly underestimated problem in diabetes patients.

## Background

Although skeletal disorders in patients with diabetes mellitus have been reported, there is still controversy over the risk of osteoporosis and its clinical significance in patients with diabetes mellitus [1-3]. Many studies have demonstrated osteopenia and increased fracture risk in patients with type 1 diabetes, however the evidence for this risk in type 2 diabetics is controversial [4-9]. Findings of increased BMD and body weight, coupled with older epidemiological studies suggesting no increase or even a decrease in fracture risk led to speculation that patients with type 2 diabetes could have a decreased risk of osteoporosis [10]. However, recent epidemiological and clinical studies provide substantial evidence for an increased fracture risk in patients with type 2 diabetes, despite an increased BMD or independently of BMD [3,11-20]. Diabetes mellitus type 1 and type 2 are considered clinical risk factors within the FRAX-algorithm which is a validated instrument to assess fracture probability [21].

The majority of older clinical studies focusing on diabetes mellitus and risk of osteoporosis are hampered by methodological problems, such as: relatively small sample sizes; heterogeneous study populations; and the use of different techniques and measurement sites for BMD. Within epidemiological studies, the information on diabetes status is often limited to self-reports without precise information on diabetes classification or control [1,2,20,22].

Osteoporosis and type 2 diabetes mellitus are commonly observed in the elderly population and will likely increase in the future. Therefore, there is a need for further investigation into the relationship between diabetes and osteoporosis risk, and related fractures. The aim of this study was to investigate the prevalence of osteoporosis (lumbar spine (LS) and femoral neck (FN) BMD< −2.5 SD T-score) and of fragility fractures, as well as determinants of BMD in a cohort of consecutive patients with known type 1 and type 2 diabetes mellitus. The prevalence of low BMD in patients with type 2 diabetes was compared with a population-based control group of the same age range living in the same area. Among possible determinants of LS-BMD and FN-BMD and risk of osteoporosis, we focused on the influence of diabetes-specific parameters like control of blood sugar, diabetes therapy, duration of disease and presence of vascular complications.

## Methods

### Patients

Diabetes group: A total of 398 patients with diabetes mellitus, comprising 155 patients with type 1 diabetes and 243 with type 2 diabetes were recruited consecutively from the outpatient clinic at the University of Heidelberg. Recruitment was performed as part of a cross-sectional, single-center study, performed in the Heidelberg study center in connection with the European Vertebral Osteoporosis Study (EVOS), which was a multicenter study to determine possible risk factors for osteoporosis [23,24]. Data collection was performed during routine follow up visits for the control of diabetes between 12/1997–12/1999.

Control group: Participants in the EVOS within the Heidelberg study center without a history of diabetes mellitus were used as a control group. These were a suitable control group for our patients with type 2 diabetes because they were a randomly selected population-based sample living in the same region with the same age distribution as the patient group. This control group comprised 255 men and 249 women [25,26].

Ethical approval was obtained from the Ethics Committee of the Heidelberg University for the EVOS and this additional study, including recruitment of diabetic patients from our outpatient department. All participants and patients gave written informed consent.

### Methods

Diabetes ascertainment and classification of diabetes type:

Diagnosis of type 1 or type 2 diabetes was based on a review of the patients’ medical records within the outpatient clinic of the Department of Internal Medicine, Endocrinology and Metabolism, University Hospital Heidelberg. The classification of type 1 diabetes was based on: hyperglycemia and insulin deficiency requiring insulin treatment; clinical course of disease with diabetic coma or other clinical manifestations; and, in most patients, on additional laboratory findings (low insulin and low C-peptide serum levels and positive GAD antibodies). There were 16/398 consecutive patients whose disease was classified as type 1 diabetes within the clinical records, who did not need insulin treatment at the time of the study (either patients with recently diagnosed diabetes or patients with longer duration of diabetes who might be misclassified as type 1). These 16 patients without need of insulin treatment were excluded from the present study. Therefore, the final study cohort comprised 139 patients (71 men, 68 women) with type 1 diabetes.

Type 2 diabetes was defined by hyperglycemia (i.e., two consecutive fasting glucose levels ≥ 7.0 mmol/dl (>100 mg/dl)) in combination with insulin resistance (obesity). Furthermore, patients who required medication typically used for treatment of hyperglycemia in type 2 diabetes (e.g. metformin) were classified as type 2 diabetics. Additional laboratory tests (insulin or C-peptide levels or GAD-antibodies) were performed only in those patients who could not be unequivocally classified by clinical parameters.

All diabetic patients were subjected to the standardized questionnaire on general risk factors for osteoporosis and for fragility fractures developed for the EVOS [26,27]. The fracture related questions were detailed with respect to location (vertebral, hip, wrist, rib and other fractures), fracture occurrence (year) and trauma severity. A fracture occurring spontaneously or after a fall from standing position was defined as a low trauma fracture. Fractures occurring after a fall from a higher position or caused by other traumatic events were classified as traumatic fractures and excluded from the analysis. Comorbidities and co-medications were assessed through a standardized questionnaire examining the following parameters: surgical therapies (gastric, intestinal, thyroid or parathyroid surgery; ovariectomy); rheumatoid arthritis; hyperthyroidism; hyperparathyroidism; hypercortisolism; chronic liver diseases; chronic gastrointestinal diseases; chronic renal diseases; nephrolithiasis; chronic lung diseases and asthma. Co-medication taken for longer than 3 months was recorded for: glucocorticoids; antacids; diuretic medications; and hormone replacement therapy.

Furthermore, standardized questions on the history of diabetes were included in the questionnaire (age at diagnosis of diabetes, diabetes specific therapy: diet, oral antidiabetics including metformin, sulfonylurea, insulin).

We evaluated data from patient records regarding microvascular and/or macrovascular complications. All respective clinical investigations were performed during the routine care of the patients within the diabetes department. With respect to microvascular complications, the presence of diabetic nephropathy was evaluated by measurement of serum creatinine and albumin excretion in morning spot urine by standardized laboratory methods (albuminuria <20 mg/l normal; 20–200 mg/l microalbuminuria, >200 mg/l macroalbuminuria). Data on diabetic retinopathy were collected from written reports from ophthalmologists and classified as normal or pathological. The ophthalmologists did not use a standardized protocol for classification of retinopathy at the time when the study was performed. The pathological findings included: maculopathy; proliferative retinopathy; vitreous hemorrhage; and nonproliferative retinopathy. Furthermore, we recorded whether laser therapy was performed. The presence of polyneuropathy was determined by clinical investigation (measurement of vibration). Macrovascular complications (coronary heart disease, myocardial infarction, stroke, peripheral arterial disease) were determined by reviewing patient records or, in cases where no positive history had been recorded, by screening for cardiological abnormalities (exercise test, echocardiography). During clinical follow up, the following tests were performed on all patients: blood pressure measurement; detection of peripheral pulse status; inspection of feet; a neurological evaluation; and blood tests (blood sugar, HbA1c, and lipid levels; renal function). Blood samples were taken from all patients and stored at −80°C.

Bone mineral density (BMD) was measured at the LS (L2–L4, LS) and at the FN by dual-energy x-ray absorptiometry (DXA) using a Hologic 4500 bone densitometer. Reference data provided by Hologic for Caucasian populations were used to compare the patients’ measurements with age- and sex-matched normal BMD data and to calculate T-scores, according to the WHO criteria [osteoporosis (t-score < −2.5 SD), osteopenia (t-score from −1 to −2.5 SD) and normal (t-score > −1 SD)] [28].

### Statistical analysis

Descriptive statistics for continuous variables included mean, median and standard deviation, categorical variables were reported with absolute and relative frequencies. The distribution of BMD was reported, stratified by gender and diabetes type, for absolute measurements and T-scores. To investigate the factors underlying the respective differences between the type 1 and type 2 diabetes groups, we used analysis of covariance (ANCOVA), with age, body mass index (BMI) and disease duration as covariates, to calculate adjusted BMD values and reported the respective least square means (LSMEANS) ± standard error (SE) for the subgroups. Pearson correlation analyses were used to assess the univariate relationship between BMD and risk factors. In addition, multiple linear regression analysis was performed to evaluate determinants of BMD, analyzed separately for type of diabetes. Age, gender, and BMI were included in the model as known predictors of BMD, and in addition, diabetic specific parameters (duration of diabetes, HbA1c level and presence of micro- or macrovascular complications) were considered. Logistic regression models were used to assess determinants of osteoporosis defined by T-score < −2.5 SD. Between-group differences were tested using t-test or ANCOVA. The difference in prevalence rates of osteoporosis between the LS-BMD and FN-BMD were analyzed using the McNemar test. Due to the low prevalence of fragility fractures, all analyses referring to fractures must be considered as descriptive and therefore no multivariate analysis on fracture determinants was performed. The level of significance was set to 5%. All statistical calculations were carried out using SAS version 9.1.

## Results

**Figure 1:**
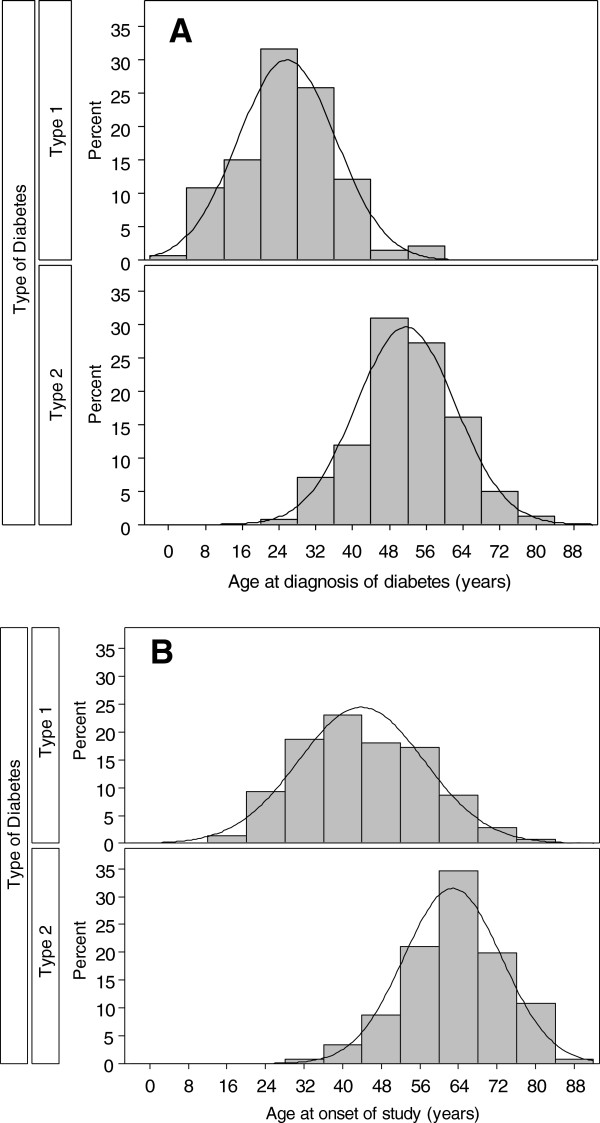
Distribution of age at time of diagnosis of diabetes subgrouped by type 1 and type 2 diabetes (A) and distribution of age at time of study performance (B).

Clinical characteristics of the patients with diabetes mellitus and the control group are described in Table 1 according to diabetes type and gender. At the time of this study, patients with type 1 diabetes were approximately 20 years younger than those with type 2 diabetes (Table 1, Figure 1B) and the BMI was significantly lower in type 1 than type 2 patients (Table 1, p < 0.0001). Type 1 diabetes was diagnosed at a younger age than type 2 diabetes, with around 15% of patients diagnosed during childhood (before 12 years of age), presumably before the onset of puberty (Figure 1A). The time since diagnosis (mean duration) of diabetes mellitus was longer in patients with type 1 diabetes than in those with type 2 diabetes (p < 0.001). Macrovascular complications including coronary heart disease, cerebrovascular complications, and peripheral artery disease as well as major comorbidities, such as hypertension, were more likely to be present in patients with type 2 diabetes than in those with type 1 diabetes. The presence of microvascular complications was not uniformly distributed: polyneuropathy was more frequent in type 2 than in type 1 diabetes, while retinopathy was more frequent in type 1 diabetes – especially the more severe forms of retinopathy (proliferative changes found in 17/137 (12.4%) type 1 diabetes patients compared with 13/237 (5.5%) in type 2 diabetes patients); no clear differences were seen for nephropathy.

**Table 1:** Characteristics of patients and control group

	**Type 1 diabetes mellitus (n = 139)**		**Type 2 diabetes mellitus (n = 243)**		**Control group (n = 504)**	
	**Men (n = 71)**	**Women (n = 68)**	**Men (n = 115)**	**Women (n = 128)**	**Men (n = 255)**	**Women (n = 249)**
	**Mean ± SD (Min. - max.)**	**Mean ± SD (Min. - max.)**	**Mean ± SD (Min. - max.)**	**Mean ± SD (Min. - max.)**	**Mean ± SD (Min. - max.)**	**Mean ± SD (Min. - max.)**
Age (years)	42.0 ± 12.9*	45.8 ± 13.0*	62.7 ± 8.5	62.9 ± 8.5	64.9 ± 8.4#	64.1 ± 8.0
	(17–42)	(22–79)	(37–83)	(28–87)	(51–82)	(51–81)
BMI (g/cm^2^)	25.2 ± 3.3*	24.6 ± 2.9*	28.9 ± 4.5	29.7 ± 5.3	27.6 ± 3.5##	26.9 ± 4.5##
	(15.9–24.8)	(18.9–32.0)	(21.6–47.8)	(18.9–47.3)	(19–45)	(16.8–42.1)
Age at menopause	-	*(n = 13)*	-	*(n = 84)*	*-*	*(n = 198)*
		41.4 ± 6.2		46.6 ± 7.2		49.9 ± 4.7
		(30–52)		(27–58)		(32–60)
Systolic blood pressure	127.8 ± 18.6	123.0 ± 17.2	136.8 ± 19.4	135.6 ± 21.1		
	(85–175)	(90–125)	(100–225)	(80–190)		
Diastolic blood pressure	74.9 ± 10.7	71.4 ± 10.2	76.2 ± 11.2	73.2 ± 11.9		
	(45–75)	(55–95)	(50.0–110.)	(45–110)		
**Diabetes specific parameters**						
Age at first diagnosis of diabetes	26.4 ± 10.2*	25.1 ± 11.1*	51.9 ± 9.4	51.3 ± 11.9		
	(8–53)	(1–55)	(31–75)	(20–80)		
Duration of diabetes (years)	15.6 ± 12.0**	20.7 ± 12.2*	12 ± 9	11 ± 8		
	(0.2–44.0)	(0.4–46.2)	(0–34)	(0–33)		
HbA1c	7.09 ± 1.17	7.15 ± 1.07	7.22 ± 1.34	7.26 ± 1.19		
	(5.3–10.4)	(4.7–10.4)	(4.8–11.4)	(4.9–11.1)		
**Diabetes therapy**	**n (%)**	**n (%)**	**n (%)**	**n (%)**		
Diet or oral antidiabetics			50 (43.5)	59 (46.1)		
Insulin	71 100.0)	68 (100.0)	65 (56.5)	69 (53.9)		
**Microvascular complications**						
**Retinopathy**	*(n = 69)*	*(n = 68)*	*(n = 111)*	*(n = 126)*		
No	41 (59.4)	42 (61.8)	86 (77.5)	86 (68.2)		
Yes	28 (40.6)	26 (38.2)	25 (22.5)	40 (31.8)		
**Polyneuropathy**	*(n = 70)*	*(n = 67)*	*(n = 115)*	*(n = 128)*		
No	49 (70)	47 (70.2)	48 (41.7)	63 (49.2)		
Yes	21 (30)	20 (29.8)	67 (58.3)	65 (50.8)		
**Nephropathy**	*(n = 71)*	*(n = 68)*	*(n = 106)*	*(n = 116)*		
Normal	50 (70.4)	55 (80.9)	61 (57.6)	81 (69.8)		
Microalbuminuria	15 (21.1)	12 (17.7)	35 (33.0)	26 (22.4)		
Macroalbuminuria	6 (8.5)	1 (1.4)	10 (9.4)	9 (7.8)		
**Macrovascular complications**						
**Coronary heart disease**	*(n = 66)*	*(n = 65)*	*(n = 108)*	*(n = 123)*		
No	58 (87.9)	60 (92.3)	77 (71.3)	100 (81.3)		
Yes	8 (12.1)	5 (7.7)	31 (28.7)	23 (18.7)		
**Myocardial infarction**	6 (8.5)	2 (2.9)	24 (20.9)	16 (12.5)		
**Cerebrovascular disease**						
(Stroke, TIA, PRIND)	2 (2.9)	0 (0)	6 (5.2)	7 (5.5)		
**Peripheral artery disease**	*(n = 68)*	*(n = 68)*	*(n = 111)*	*(n = 124)*		
No	62 (91.2)	63 (92.6)	91 (82.0)	105 (84.7)		
Yes	6 (8.8)	5 (7.4)	20 (18.0)	19 (15.3)		
**Hypertension**						
yes	27 (38.0)	21 (30.9)	83 (72.2)	97 (75.8)		

Comparison between type 1 and type 2 diabetes groups by gender: *p < 0.0001; **p < 0.001.

Comparison between type 2 diabetes group with control group by gender: #p < 0.02, ##p < 0.006.

TIA (transient ischemic attack); PRIND (prolonged ischemic neurological deficit).

The distribution of comorbidities and co-medications is summarized in the Additional file 1: Table S1. Among patients with type 1 diabetes the majority of assessed comorbidities were rarely reported (prevalence less than 5%). However, hyperthyroidism, rheumatoid arthritis and chronic lung disease were each reported in up to 10% of the women. For both men and women with type 2 diabetes, a history of rheumatoid arthritis, hyperthyroidism, nephropathy or chronic lung disease was reported in 5–15% of patients. Use of glucocorticoids was reported in 6–12.5% and antacids were used by 6.5–18.6% of patients.

### Distribution of BMD in patients with type 1 and type 2 diabetes

**Figure 2:**
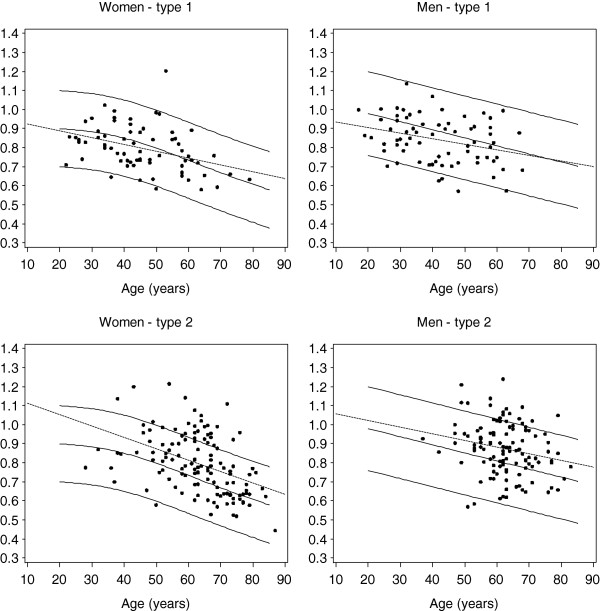
Distribution of femoral neck bone mineral density (BMD) subgrouped according to type of diabetes and gender in comparison to the normal distribution (Hologic reference population, mean ± 2 SD).

Figure 2 illustrates the age and sex specific distributions of FN-BMD in patients with type 1 and type 2 diabetes in relation to the respective reference distributions. Men and women with type 1 diabetes had BMD values usually within the reference range and the linear regression lines fitted to the data did not significantly differ from the mean reference curve values. In contrast, patients with type 2 diabetes had significantly higher mean FN-BMD compared with the mean reference values. Similar distributions were found for LS-BMD in all subgroups (data not shown).

The comparison of BMD values between patients with type 1 and type 2 diabetes mellitus and the control group is shown, stratified by gender, in Tables 2 and 3 (absolute measurements, T-scores and adjusted BMD values). Age-adjusted mean BMD at the FN was significantly lower in men and women with type 1 diabetes compared with those with type 2 diabetes (p = 0.0004 and p = 0004). A similar trend was found for BMD at the LS, while the differences were smaller and reached significance only in women (p = 0.02). However, when BMD values were adjusted for age, BMI and duration of disease, the adjusted values were not different between patients with type 1 and type 2 diabetes. In contrast, in both male and female patients with type 2 diabetes, the adjusted BMD values remained significantly higher at the FN and LS compared with the age-matched control group.

**Table 2:** Distribution of bone mineral density (BMD) in patients with type 1 and type 2 diabetes mellitus and the control group by gender (men)

	**Men**				
	**Type 1 n = 71**	**Type 2 n = 115**	**Control n = 255**	**Type 1 vs. type 2**	**Type 2 vs. control**
**BMD**	Mean ± SD	Mean ± SD	Mean ± SD	p-value	p-value
Lumbar BMD (g/cm^2^)	1.05 ± 0. 14	1.08 ± 0.18	1.03 ± 0.20	0.263	0.03
Femoral Neck BMD (g/cm^2^)	0.84 ± 0.12	0.88 ± 0.15	0.81 ± 0.13	0.079	0.0001
T-Score (SD) Lumbar BMD	−0.56 ± 1.34	−0.31 ± 1.61	−0.76 ± 1.78	0.268	0.017
T-Score (SD) Femoral Neck BMD	−1.20 ± 1.11	−0.90 ± 1.39	−1.55 ± 1.24	0.104	0.0001
Adjusted BMD*	LSMEAN ± SE	LSMEAN ± SE	LSMEAN ± SE		
Lumbar BMD (g/cm^2^)	1.05 ± 0.03	1. 08 ± 0.02	1.03 ± 0.01	0.453	0.027
Femoral Neck (g/cm^2^)	0.83 ± 0.02	0. 88 ± 0.02	0.81 ± 0.01	0.11	0.0011
**WHO-criteria**	**% (n)**	**% (n)**	**% (n)**		
**Lumbar BMD T-score**					
<−2.5 SD (Osteoporosis)	5.6 (4)	6.1 (7)	14.9 (38)		
−2.5 SD – −1.0 SD (Osteopenia)	31.0 (22)	30.4 (35)	32.9 (84)		
>−1.0 SD (Normal)	63.4 (45)	63.5 (73)	52.2 (133)		
**WHO-criteria**					
**Femoral Neck BMD T-score**					
<−2.5 SD (Osteoporosis)	9.9 (7)	13.0 (15)	21.2 (54)		
−2.5 SD – −1.0 SD (Osteopenia)	45.1 (32)	35.7 (41)	49.8 (127)		
>−1.0 SD (Normal)	45.1 (32)	51.3 (59)	29.0 (74)		

SD = Standard deviation.

LSMEAN = Least Square Mean (age adjusted BMD-values), SE = Standard Error.

*Adjusted for age, duration of diabetes and BMI.

**Table 3:** Distribution of bone mineral density (BMD) in patients with type 1 and type 2 diabetes mellitus and the control group by gender (women)

	**Women**				
	**Type 1 n = 68**	**Type 2 n = 128**	**Control n = 249**	**Type 1 vs. type 2**	**Type 2 vs. control**
**BMD**	Mean ± SD	Mean ± SD	Mean ± SD	p-value	p-value
Lumbar BMD (g/cm^2^)	1.03 ± 0.15	1.02 ± 0.17	0.90 ± 0.18	0.733	0.0001
Femoral Neck BMD (g/cm^2^)	0.80 ± 0.12	0.79 ± 0.16	0.70 ± 0.12	0.940	0.0001
T-Score (SD) Lumbar BMD	−0.45 ± 1.37	−0.46 ± 1.56	−1.65 ± 1.53	0.974	0.0001
T-Score (SD) Femoral Neck BMD	−1.01 ± 1.15	−0.95 ± 1.57	−1.96 ± 1.13	0.772	0.0001
Adjusted BMD*	LSMEAN ± SE	LSMEAN ± SE	LSMEAN ± SE		
Lumbar BMD (g/cm^2^)	1.01 ± 0.03	1.05 ± 0.01	0.91 ± 0.01	0.337	0.0001
Femoral Neck (g/cm^2^)	0.78 ± 0.02	0.80 ± 0.01	0.71 ± 0.01	0.560	0.0001
**WHO-criteria**	**% (n)**	**% (n)**	**% (n)**		
**Lumbar BMD T-score**					
<−2.5 SD (Osteoporosis)	5.9 (4)	9.4 (12)	26.9 (67)		
−2.5 SD – −1.0 SD (Osteopenia)	29.4 (20)	27.3 (35)	42.6 (108)		
>−1.0 SD (Normal)	64.7 (44)	63.3 (81)	30.5 (76)		
**WHO-criteria**					
**Femoral Neck BMD T-score**					
<−2.5 SD (Osteoporosis)	10.3 (7)	21.9 (28)	32.1 (80)		
−2.5 SD – −1.0 SD (Osteopenia)	41.2 (28)	31.2 (40)	45.8 (114)		
>−1.0 SD (Normal)	48.5 (33)	46.9 (60)	22.1 (55)		

SD = Standard deviation.

LSMEAN = Least Square Mean (age adjusted BMD-values), SE = Standard Error.

*Adjusted for age, duration of diabetes and BMI.

### Prevalence of osteoporosis in patients with diabetes compared with the control group

The prevalence of osteoporosis, osteopenia and normal BMD in patients with diabetes and the control group based on femoral BMD and LS-BMD measurements is summarized in Tables 2 and 3, stratified by gender. No difference was observed between men and women with type 1 diabetes for the prevalence of osteoporosis at the FN (9.9% of men and 10.3% of women). However, women with type 2 diabetes had a higher prevalence (21.9%) than men with type 2 diabetes (13.0%). The respective prevalence of osteoporosis at the LS was 5.6% in men and 5.9% in women with type 1 diabetes and 6.1% in men and 9.4% in women with type 2 diabetes. The proportion of patients with osteoporosis at the FN (T-score < −2.5 SD) was higher compared with the proportion of patients with osteoporosis at the LS in all groups, however this difference only reached statistical significance in patients with type 2 diabetes (p = 0.03 in men, p = 0.001 in women).

The prevalence of osteoporosis was significantly lower at both measurement sites in patients with type 2 diabetes in comparison with the age-matched, population-based control group (LS-BMD: men 14.9%, women 26.9%; FN-BMD: men 21.2%, women 32.1%). The risk of having osteoporosis according to BMD criteria was approximately halved in patients with type 2 diabetes compared with the control group [LS-BMD Odds ratio (95% CI): men 0.36 (0.16–0.83); women 0.28 (0.14–0.53) and FN-BMD Odds ratio (95% CI): men 0.52 (0.28–0.97); women 0.59 (0.36–0.97)].

### Determinants of BMD in patients with type 1 and type 2 diabetes

We observed a significant and positive correlation between BMI and FN-BMD. The correlation was more pronounced in men and women with type 2 diabetes (r = 0.37, p < 0.0001 and r = 0.44, p <0.0001) than men and women with type 1 diabetes (r = 0.24, p <0.04 and r = 0.20, p <0.09). Duration of diabetes and FN-BMD were significantly and negatively correlated in women with type 1 diabetes (r = −0.34, p < 0.005) but not in men with type 1 diabetes (r = −0.18, p < 0.13) and not in patients with type 2 diabetes (r = −0.11, p = 0.2 in women, r = −0.15, p = 0.1 in men). HbA1c levels were not correlated with BMD in any subgroup (type 1 diabetes: women: r = −0.03, men: r = −0.03; type 2 diabetes: women: r = −0.076, men: r = −0.07). Furthermore, there was no difference in BMD between women with type 2 diabetes treated with diet, oral antidiabetics or insulin therapy, but BMD was lower in men with type 2 diabetes undergoing insulin treatment (LSMEAN ± SE; FN-BMD 0.86 ± 0.02 g/cm^2^; LS-BMD 1.035 ± 0.02 g/cm^2^) compared with those treated with other therapies (FN-BMD 0.91 ± 0.02 g/cm^2^; LS-BMD 1.13 ± 0.02 g/cm^2^) (p = 0.02; p = 0.004). Age-adjusted FN-BMD was compared with respect to the presence of micro- or macrovascular complications subgrouped by diabetes type and gender. For women with type 1 diabetes, there was a tendency for lower BMD values in patients with coronary heart disease than in those without coronary heart disease (FN-BMD LSMEAN 0.73 ± 0.05 g/cm^2^ vs. 0.80 ± 0.013 g/cm^2^; p = 0.13). The respective differences were only marginal within the other subgroups. None of the other micro- and macrovascular complications (retinopathy, polyneuropathy, nephropathy, peripheral artery disease), showed a relationship to BMD (data not shown, available on request).

Multiple linear regression analyses were performed to identify determinants of LS-BMD and FN-BMD in patients with type 1 and type 2 diabetes (detailed results see Table 4). In patients with type 1 diabetes, only age and BMI were significantly associated with LS-BMD and FN-BMD, while diabetes-specific variables (duration of diabetes, HbA1c level, presence of vascular complications) showed no significant influence. In patients with type 2 diabetes, LS-BMD was significantly determined by gender, age, BMI and duration of diabetes, while FN-BMD was dependent on gender, age and BMI. Similar results were found within a multivariable logistic regression analysis to identify determinants of a FN-BMD below a T-score of −2.5 SD. Age was the only significant predictor of a FN-BMD below T-score of −2.5 SD in patients with type 1 diabetes, while in type 2 diabetes, age and BMI were significantly associated with a low BMD.

**Table 4:** Multiple linear regression analyses of determinants of bone mineral density (BMD) in patients with type 1 and type 2 diabetes

	**Diabetes mellitus type 1**			
	**Dependent variable: lumbar BMD**		**Dependent variable: femoral neck BMD**	
	**Parameter estimate; SE**	**p-value**	**Parameter estimate; SE**	**p-value**
Gender (women vs. men)	0.0050; 0.02	0.84	−0.019; 0.02	0.32
Age	−0.0025; 0.001	0.04	−0.003; 0.0009	0.0004
Duration of diabetes	−0.0009; 0.001	0.51	−0.0009; 0.001	0.51
BMI	0.013; 0.004	0.001	0.011; 0.003	0.0003
Presence of micro- or macrovascular complications	0.004; 0.03	0.88	0.022; 0.02	0.38
HbA1c	−0.02; 0.01	0.08	−0.008; 0.008	0.37
	**Diabetes mellitus type 2**			
	**Dependent variable: lumbar BMD**		**Dependent variable: femoral neck BMD**	
	**Parameter estimate; SE**	**p-value**	**Parameter estimate; SE**	**p-value**
Gender (women vs. men)	−0.05; 0.02	0.02	−0.079; 0.02	0.0001
Age	−0.003; 0.001	0.02	−0.003; 0.001	0.001
Duration of diabetes	0.004; 0.001	0.01	0.00047; 0.001	0.704
BMI	0.007; 0.002	0.002	0.010; 0.002	0.0001
Presence of micro- or macrovascular complications	−0.0006; 0.03	0.98	−0.016; 0.02	0.51
HbA1c	−0.0126; 0.008	0.15	−0.01; 0.007	0.14

### Fracture history in type 1 and type 2 diabetes

Information on fracture history was complete in 85% of our diabetic patients (n = 325). Traumatic fractures were reported in 12 patients (3 rib fractures, 4 wrist and 7 other fractures (note some patients had multiple fractures)) - these fractures were excluded from further analysis. At least one low trauma fracture was reported for 17 patients after diabetes was confirmed. The mean time between diagnosis of diabetes and fracture occurrence was 10.3 ± 8.5 years in patients with type 1 and 5.8 ± 6.7 years in patients with type 2 diabetes. In these 17 patients, a total of 22 low trauma fractures were reported and distributed as follows: two vertebral, one hip, four rib, four wrist, and 11 other fractures. The overall prevalence of persons with a history of low trauma fracture (17/325, 5.2%) did not significantly differ according to gender or type of diabetes [type 1 diabetes: men 4/63 (6.4%); women 4/63 (6.4%); type 2 diabetes: men 3/94 (3.2%); women 6/105 (5.7%)].

**Figure 3:**
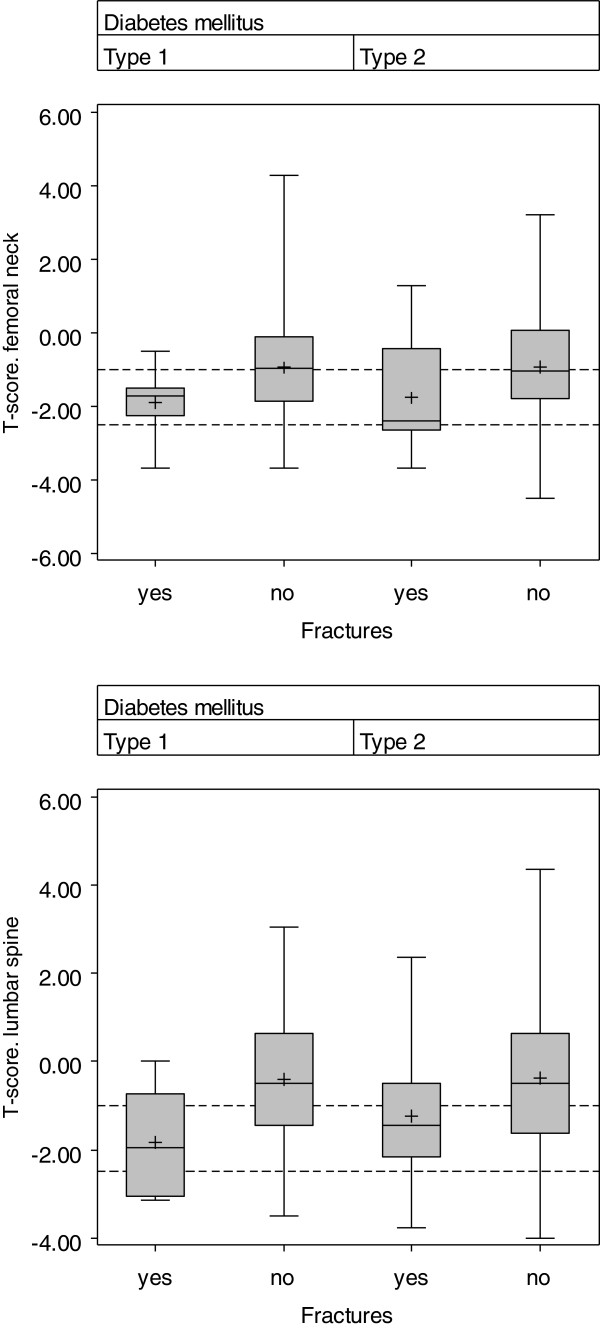
Distribution of bone mineral density (BMD) (Box-plots) in patients with and without insufficiency fractures subgrouped by type of diabetes mellitus; T-score femoral neck BMD (upper part), T-score lumbar spine BMD (lower part).

The distribution of FN-BMD and LS-BMD (T-scores) subgrouped into patients with and without fractures by type of diabetes is shown in Figure 3. There was a trend for lower BMD values in patients with fractures compared with those without fractures in all subgroups, however BMD showed a remarkable overlap between patients with fractures and those without and most patients with fractures had T-scores above −2.5 SD. In patients with type 1 diabetes, LS-BMD and FN-BMD were significantly lower in those with fractures compared with those without fractures (LS-BMD p <0.02 and FN-BMD p < 0.03) while in patients with type 2 diabetes there was a similar trend which did not reach statistical significance (LS-BMD p < 0.2 and FN-BMD p < 0.2). Age, BMI, diabetes duration and HbA1c levels were not different between patients with and without fractures, subgrouped by diabetes type. The occurrence of fractures was not significantly different between men and women and showed no association with the presence of vascular complications in patients with type 1 and type 2 diabetes.

## Discussion

### Distribution of BMD in patients with type 1 and type 2 diabetes

In summary, our study shows that the prevalence of low BMD and fractures was similar in patients with type 1 and type 2 diabetes, although patients with type 1 were about 20 years younger than those with type 2 diabetes. The comparison of BMD distribution with a population-based control group suggests a lower risk of osteoporosis in type 2 diabetes. However, the prevalence of fragility fractures was not different between patients with type 1 (6.4%) and type 2 diabetes (4.5%). In both diabetic groups, we found lower BMD values at the FN than at the LS. Determinants of BMD were body mass index and age, but not diabetes specific parameters including diabetes control (HbA1c level), duration and vascular complications. Occurrence of low trauma fracture was related to lower BMD values, however most patients with fractures had BMD-values above a threshold of T-score of −2.5 SD.

Our finding of lower FN-BMD than LS-BMD in patients with diabetes is in agreement with some studies, while others did not find such a difference [6,9,29-32]. Most studies focus on either type 1 or 2 diabetes, or do not precisely define the diabetes type at all and, thus, the assessment of a cohort of consecutive patients of both diabetes types is unique to our study. The increased bone loss at the FN compared with the LS-BMD suggests that osteoporosis in diabetic patients preferentially develops within the appendicular skeleton with predominantly cortical bone. One possible explanation could be the presence of secondary hyperparathyroidism due to vitamin D deficiency, as PTH causes predominantly cortical bone loss. However, this needs further investigation. Another explanation for increased LS-BMD values in patients with type 2 diabetes could be an artificially high determination of BMD due to degenerative changes and diffuse idiopathic skeletal hyperostosis, frequently found in such patients [33-36]. Although the reasons for the divergent distribution of bone mass at the FN and LS cannot be clarified by our study, this finding is of practical relevance and suggests that evaluation of osteoporosis risk in diabetic patients should include both measurement sites and not rely on one. Ideally, measurement of the forearm with predominantly cortical bone should also be included.

The increased LS-BMD and FN-BMD found in our patients with type 2 diabetes in comparison with a population-based control group confirms similar findings from epidemiological studies. There is growing evidence for an increased risk of fractures in patients with diabetes mellitus despite normal or even high BMD values [3,16,37]. Although our study size was not powered for the assessment of fracture determinants, and the number of patients with fragility fracture was relatively low, it was evident that patients with fractures had significantly lower (type 1 diabetes) or a trend towards lower (type 2 diabetes) BMD values than patients without fractures. However, BMD values were above the T-score of −2.5 SD in most patients with fractures. This suggests that there is an association between fracture risk and BMD in diabetic patients, but the fracture threshold is higher than that associated with non-diabetic populations (T-score < −2.5 SD). Our data support findings of a recent epidemiological study on the association between BMD, FRAX-score and fracture risk in older adults with diabetes [22], showing that the level for increased fracture risk seems to be about 0.5 SD T-Score higher than in nondiabetics. In contrast, Yamamoto et al., found no relationship between fracture risk and BMD in diabetic patients, although the rate of fractures was rather high (30%) in this study, suggesting some selection bias [19]. Both studies [19,22] focus on patients with type 2 diabetes, while association studies on fracture risk and BMD in patients with type 1 diabetes are rare [3]. The relatively high rate of low trauma fractures and of low BMD found in our patients with type 1 diabetes mellitus with a median age of 45 years, underlines the clinical importance of changes in bone metabolism as a complication associated with type 1 diabetes mellitus that is probably underestimated and deserves more consideration during patient care.

### Predictors of BMD and fractures in type 1 and 2 diabetes

The assessment of diabetes specific parameters and osteoporosis only showed some weak relationships, and our data did not permit the reliable prediction of the risk of low BMD (T-score < −2.5 SD) or the occurrence of low trauma fractures from clinical parameters. There was a weak negative correlation between diabetes duration and FN-BMD in type 1 but not in type 2 diabetes. A lack of insulin is speculated to exert a negative influence on bone formation resulting in a decreased peak bone mass in patients with type 1 diabetes, as the disease often starts at a time when peak bone mass is not yet achieved [38,39]. About 15% of our patients with type 1 diabetes were diagnosed before puberty. Further longitudinal studies are required to assess the influence of diabetes onset before or after puberty on the development of peak bone mass and risk of osteoporosis during later life. In patients with type 2 diabetes the type of therapy (diet, oral antidiabetics, insulin) was not related to the risk of osteoporosis in women, while men with insulin therapy had slightly lower BMD values. None of our patients were treated with thiazolidinedione, so this known risk factor for osteoporosis was not relevant in our analysis.

We investigated the contribution of several comorbidities and co-medications known to influence bone health – the prevalence was low (<5%) for most of these factors within our patient groups, with a number reported between 5 and 15%. However, the frequencies of affected patients were not different between subgroups. As the number of affected patients was so low, it was not feasible to consider these aspects within multivariate analysis.

One aim of our study was to assess the relationship between the occurrence of micro- and macrovascular complications and the risk of osteoporosis. We found no significant relationship between vascular complications and BMD. However, in female type 1 diabetes patients, there was a trend for lower FN-BMD in patients with coronary heart disease than in those without. This supports the hypothesis that vascular changes and complications influence bone metabolism and contribute to the development of osteoporosis as long suggested by histopathological studies of bone biopsies [40] and from studies showing an association between atherosclerosis and low bone mineral density [41-45].

In a multivariate model, only age and BMI were significant predictors of BMD in type 1 diabetes, while in type 2 diabetes, gender was also predictive for BMD, probably reflecting the higher prevalence of postmenopausal status within this subgroup. Neither gender, nor parameters of disease control (such as HbA1c levels, therapy regimen (oral antidiabetics vs. insulin), duration and complications of diabetes), predicted BMD. Schwarz et al., had shown that insulin treatment increased the risk of falls, which probably contributes to the increased fracture risk [46].

Due to the study size and the relatively low number of fragility fractures, our data does permit the assessment of fracture determinants by a multivariate model. In both diabetes groups, the mean BMD values were lower in patients with fragility fractures compared with those without. However, there was a remarkable overlap between patients with and without fractures, and in most patients with fractures, the BMD was above the fracture threshold of −2.5 SD T-score. Possible reasons for the increased bone fragility independent of BMD include changes in bone structure and bone quality caused by hyperglycemia and the accumulation of advanced glycated end products (AGES) of bone matrix proteins. Therefore, the longer duration of disease with concomitant longer period of potential hyperglycemia in type 1 diabetes mellitus may be associated with fracture risk independently of BMD. Furthermore, several non-skeletal factors may be related to fracture risk, such as an increased propensity of falls, secondary to diabetic complications like visual impairment or neuropathy [2,15,46].

Although most low trauma fractures recorded in our patients were not located at the skeletal sites considered typical for osteoporosis, like vertebrae or hip, they are of clinical importance as these fractures are known to account for 80% of the clinical fractures especially in younger patients, and may precede a hip fracture [47]. The association of these low trauma fractures at any site to a lower BMD in our diabetic patients supports these as being “osteoporotic” fractures. In clinical practice, the occurrence of any low trauma fracture in diabetic patients should be closely monitored, to identify those patients who are at increased risk for further fractures, and to initiate diagnostic evaluation for osteoporosis and therapy.

### Strengths and limitations

The strength of our study is the standardized evaluation of diabetic patients including a questionnaire of possible osteoporosis risk factors and BMD measurements, which applied the same regimen as a population-based study on osteoporosis (EVOS), performed within a similar time period and region, and used as a control group for the type 2 diabetes patient group. We were able to assess a relatively large, unselected cohort of consecutive patients with type 1 and type 2 diabetes with a nearly equally distributed number of men and women, allowing a comparison between the respective subgroups. In all diabetic patients, standardized documentation of diabetes-related parameters including type of diabetes, diabetes therapy, control and diabetes-related vascular complications was available, ensuring a high data quality. One limitation is the lack of an adequate control group for the type 1 diabetes patient group, due to the age difference between the population-based control group and most patients with type 1 diabetes, although this limitation was minimized through adjustment of BMD values according to age, BMI and duration of diabetes. A second limitation is the relatively small sample size of diabetic patients in this study with respect to the complex questions addressed, especially when stratified by diabetes type and gender. Thus, our findings have to be considered descriptive.

A third limitation of our study is the cross-sectional design, which restricts the assessment of causal relationships. Furthermore, data on low trauma fractures were only based on a questionnaire and patient records. Non-skeletal parameters such as the risk of falls, including muscle weakness, dizziness and cerebrovascular diseases are major determinants of fractures in addition to BMD and were not assessed within the present study. Finally, the relatively low number of patients with fragility fractures limits conclusive statistical analysis, especially regarding fracture occurrence.

## Conclusion

Our study shows a similar risk of osteoporosis in patients with type 1 diabetes based on low BMD (T-score < −2.5 SD), which was not different from the prevalence in patients with type 2 diabetes despite them being approximately 20 years older. The FN-BMD was particularly decreased; therefore evaluation of osteoporosis risk in younger patients with type 1 diabetes should include both spinal and FN-BMD-measurements.

In agreement with others, we found increased LS-BMD and FN-BMD in patients with type 2 diabetes in comparison with a non-diabetic population-based control group. There was a trend for lower BMD in diabetic patients (type 1 and 2) with osteoporotic fractures compared with those without fractures, however the fracture threshold is higher than in non-diabetic populations. Further longitudinal cohort studies are required, focusing on the risk of fractures and changes in bone metabolism in patients with diabetes. BMD measurements and the evaluation of BMD-independent risk factors for fractures should be included in the routine management of patients with diabetes mellitus because the prediction of osteoporosis solely by clinical diabetes-specific parameters was not possible. The evidence suggests that osteoporosis, and related fractures, is a clinically significant and commonly underestimated problem in patients with type 1 and type 2 diabetes mellitus.

## Competing interests

All authors (GLB, SG, TB, CSN, PN, JGS) declare that they have no competing interests.

## Authors’ contributions

GLB, CSN, JGS were responsible for the design, acquisition, analysis and interpretation of the data. TB participated in the design of the study and performed statistical analyses. SG, JGS conducted chart review of diabetic patients, CSN was responsible for the EVOS study. All authors (GLB, SG, TB, CSN, PN, JGS) participated in interpretation of the study, drafting the manuscript and approve of the final submission.

## Pre-publication history

The pre-publication history for this paper can be accessed here:

http://www.biomedcentral.com/1472-6823/14/33/prepub

## Supplementary Material

Additional file 1: Table S1 Comorbidities and co-medications. Click here for file
