# Supplementary material for: Prevalence and determinants of osteoporosis in patients with type 1 and type 2 diabetes mellitus
Source: BMC Endocr Disord. 2014 Apr 11;14:33. doi: 10.1186/1472-6823-14-33 (PMC4021186; doi:10.1186/1472-6823-14-33)
Supplement: Additional file 1: Table S1 — Comorbidities and co-medications. [file 1472-6823-14-33-S1.doc]

**Additional file 1**

**Table S1.** Comorbidities and co-medication

|  | Type 1 diabetes mellitus  (n=139) | | Type 2 diabetes mellitus  (n=243) | |
| --- | --- | --- | --- | --- |
|  | men  (n=71) | women  (n=68) | men  (n=115) | women  (n=128) |
| Comorbidities | n | n | n | n |
| **History of surgery** |  |  |  |  |
| gastric | 1 | 0 | 3 | **2** |
| intestinal | 4 | 0 | 5 | 4 |
| thyroid | 4 | 3 | 3 | 10 |
| parathyroid | 1 | 0 | 3 | 0 |
| ovariectomy (both) | - | 1 | - | 16 |
| hysterectomy | - | 15 | - | 48 |
|  |  |  |  |  |
| **History of chronic diseases** |  |  |  |  |
| rheumatoid arthritis | 4 | 6 | 12 | 13 |
| hyperthyroidism | 2 | 8 | 8 | 14 |
| hyperparathyroidism | 0 | 0 | 2 | 1 |
| liver | 2 | 1 | 8 | 4 |
| bowel / intestinal | 2 | 0 | 7 | 6 |
| renal | 1 | 2 | 2 | 4 |
| nephrolithiasis | 1 | 2 | 11 | 9 |
| pulmonary | 2 | 6 | 11 | 10 |
|  |  |  |  |  |
| **Co-medication** |  |  |  |  |
| glucocorticoids | 4 | 7 | 8 | 8 |
| antiepileptics | 0 | 3 | 2 | 2 |
| antacids | 4 | 6 | 16 | 16 |
| diuretics | 5 | 2 | 19 | 26 |
| hormone replacement | - | 13 | - | 19 |
